# ^177^Lu−SN201 nanoparticle shows superior anti−tumor efficacy over conventional cancer drugs in 4T1 orthotopic model

**DOI:** 10.1007/s10637-024-01450-2

**Published:** 2024-06-05

**Authors:** Sujinna Lekmeechai, Kristian Pietras, Oskar Axelsson

**Affiliations:** 1 Spago Nanomedical AB, Scheelevägen 22, Lund, SE-223 63 Sweden; 2 Department of Laboratory Medicine, Division of Translational Cancer Research, Lund University Cancer Centre, Medicon Village, Bldg 404, Lund, SE-223 81 Sweden

**Keywords:** Targeted radiotherapy, ^177^Lu-SN201, Cancer, Nanoparticle, Radiotherapy, ^177^Lu

## Abstract

In the current in-vivo study we demonstrate the potential of the radiolabeled nanoparticle ^177^Lu-SN201 as an effective anticancer treatment, as evidenced by significantly prolonged survival and reduced tumor burden in the aggressive, triple negative 4T1 murine breast cancer model. We show with high statistical significance that ^177^Lu-SN201 is superior at suppressing the tumor growth not only compared to vehicle but also to the commonly used cancer drugs paclitaxel, niraparib, carboplatin, and the combination of the immune checkpoint inhibitors anti PD-1 and anti-CTLA-4. The dosing of the standard drugs were based on examples in the literature where good effects have been seen in various mouse models. The treatment is reasonably well-tolerated, as indicated by clinical chemistry of liver and renal function through the measurement of glutamate pyruvate alanine aminotransferase, alanine amino transferase, blood urea nitrogen, and creatinine levels in plasma samples, despite some weight loss. Overall, ^177^Lu-SN201 presents as a promising therapeutic candidate for cancer treatment.

## Introduction

The treatment of solid cancers, at advanced stages of disease, is challenging due to their aggressive nature and inherent genomic instability, which fosters diverse tumor cell populations with distinct molecular signatures. This intratumoral heterogeneity leads to non-uniform responses to therapy and the emergence of drug-resistant subpopulations, complicating treatment strategies and resulting in limited efficacy of traditional therapeutic approaches. Solid tumors often exhibit a complex microenvironment, which hampers drug delivery and promotes resistance to therapy [1, 2]. Innovative strategies that can overcome these obstacles and deliver targeted therapies directly to tumor sites are urgently needed.

One such strategy involves exploiting the Enhanced Permeation and Retention (EPR) effect, which is a common physiological feature found in solid tumors. The EPR effect, observed across various animal models and in human patients, is a group of pathophysiological features of solid tumors leading to the intratumoral accumulation of macromolecules. Abnormalities in tumor blood vessels, and inefficient lymphatic drainage in solid tumors are the main contributors, facilitating passive tumor delivery of anticancer drugs that are optimized to pass through cancer leaky blood vessels, but not through the normal blood vessels of healthy tissue [3]. This mechanism of action is supported by our previous study on ^177^Lu-SN201 where we investigated the biodistribution and pharmacokinetics in some detail [4].

Some other innovative approaches have been published recently, one involving the use of nanoparticles for radiations sensitization [5] and one where the ^177^Lu-loaded nanoparticles were injected into the tumor, a method that is often called nanobrachytherapy [6].

Recent studies with the contrast agent SN132D, derived from the same nanoparticle platform as ^177^Lu-SN201, have demonstrated magnetic resonance imaging (MRI) contrast enhancement in breast tumors in a clinical trial [7]. These encouraging results underscore the promising clinical utility of our EPR-based nanoparticle approach in the targeting of solid tumors.

We have developed a novel polymeric nanoparticle, termed ^177^Lu-SN201, designed to exploit the EPR effect for targeted delivery of the therapeutic radionuclide, ^177^Lu, to solid tumors. It has recently entered a phase 1 study in cancer patients [8]. By optimizing the size and surface properties of bioinert polymeric nanoparticles, we aim to overcome the limitations associated with traditional systemic chemotherapy and deliver therapeutic doses of radiation to tumor sites via the EPR effect. The efficacy of this nanoparticle has previously been shown in the triple negative breast cancer (TNBC) model 4T1 [4]. However, the subcutaneous version of the model, while informative, turned out to be less than ideal because of a large incidence of ulcerations, leading to loss of animals, which makes the results more difficult to interpret. The orthotopic version of the model, where 4T1 cells are implanted in the mammary fat pad, is less prone to ulceration and was used for the experiments reported below. The orthotopic model appeared to be superior, compared to subcutaneous model.

In this study, we present a comparison of the efficacy of ^177^Lu-SN201 with commonly used anticancer drugs in the orthotopic 4T1 TNBC model. These agents are commonly used drugs in the treatment of TNBC and have different modes of action [9]: paclitaxel, niraparib, carboplatin, and the combination of anti PD-1 and anti-CTLA-4, which will be referred to as immune checkpoint inhibitors (ICI) for simplicity throughout this manuscript [10]. Paclitaxel, a microtubule-stabilizing taxane, disrupts mitotic spindle function, leading to cell cycle arrest and apoptosis [11]. ICI is an approach that aims to unleash the immune system’s ability to recognize and eradicate cancer cells by overcoming the immune evasion mechanisms employed by tumor cells [10]. Carboplatin, a platinum-based chemotherapeutic agent, induces DNA crosslinking and subsequent apoptosis in rapidly dividing cancer cells [12]. Niraparib, a poly (ADP-ribose) polymerase (PARP) inhibitor, disrupts DNA repair mechanisms, leading to cancer cell death [13]. In this study, we will add to the growing body of evidence that the EPR effect is a useful tumor targeting mechanism by presenting results indicating that ^177^Lu-SN201 displays superior efficacy compared to these drugs.

## Materials and methods

### Cell culture

The murine mammary carcinoma cell line 4T1 was obtained from ATCC (American Type Culture Collection, USA). The 4T1 cell line was maintained at 37 °C in a humidified atmosphere (5% CO_2_, 95% air) using RPMI 1640 medium supplemented with 10 mM Hepes, 2.5 g/L glucose, 1 mM sodium pyruvate, and 10% fetal bovine serum. Cell number and viability were assessed by trypan blue exclusion assay using a 0.25% trypan blue solution.

### Animal husbandry

All animal procedures conducted in this study (Ethical protocol: #266,110) were approved by the Institutional Animal Care and Use Committee in compliance with Greek authorities (Protocol number: 22,066). The animal facility where the experiments were conducted is accredited by the Greek National Committee for Laboratory Animals and under the License EL 25 BIOexp 045.

Mice were maintained under controlled environmental conditions, with temperatures set at 22 ± 2 °C, humidity maintained at 55 ± 10%, and subjected to a 12-hour light-dark cycle. Food and water were provided ad libitum throughout the study period.

### Mouse 4T1 orthotopic model

One hundred and eighty healthy female BALB/c (BALB/cByJ) mice, aged 7–8 weeks, were obtained from Charles River and acclimatized for one week. Tumor inoculation was performed under anesthesia using isoflurane. The skin over the lateral thorax was incised to expose the mammary fat pad (MFP) before the injection of 1 × 10^5^ 4T1 breast cells, suspended in 50 µL RPMI 1640 medium, followed by suturing. Tumors were allowed to grow until the volume reached 1000 mm^3^ prior to the beginning of anti-cancer treatment.

### Cancer treatment and efficacy assessment

Upon reaching palpable size (100 mm^3^), the mice were randomly allocated into 9 groups: (1) ^177^Lu-SN201 vehicle (Ringer’s buffer, Fresenius Kabi), also serving as the ICI vehicle control), (2) Paclitaxel vehicle (10% Cremophor and 10% ethanol), (3) Carboplatin vehicle (5% DMSO), (4) Niraparib vehicle (10% DMSO, 40% PEG 300, 5% Tween-80, and 45% saline), (5) ^177^Lu-SN201 (Spago Nanomedical), (6) ICI (InVivoMAb anti-mouse PD-1, BioXCell, # BE0146 and InVivoMAb anti-mouse CTLA-4, BioXCell, #BE0164), (7) Paclitaxel (Selleckchem, # S1150), (8) Carboplatin (MCE, HY-17,393), (9) Niraparib (Selleckchem, # S2741). The formulation and radiolabeling of ^177^Lu-SN201 were performed as described in a previous study [4]. A dose of 4 MBq was administered intravenously on Day 1 of the treatment schedule. ICI (anti-PD-1 200 µg/mouse and anti-CTLA-4 100 µg/mouse) were dissolved in PBS and administered intraperitoneally on days 1, 4, 7, and 10 [14]. Paclitaxel at a dose of 6 mg/kg was administered intraperitoneally on days 1, 3, 5, 7, and 9 [15]. Carboplatin was administered at a dose of 100 mg/kg via intraperitoneal injection on days 1 and 8 [16]. Niraparib at a dose of 50 mg/kg was administered orally once daily from day 1 to day 10 [17]. Vehicles were administered following the same schedule as their corresponding drugs.

Tumor volume and body weight were measured throughout the monitoring period. Mice were terminated when humane endpoints were reached. Upon termination, tumors and lungs were weighed, and plasma samples were collected for further analysis.

### Clinical Chemistry

Total bilirubin, creatinine (Cre), blood urea nitrogen (BUN), alkaline phosphatase (ALP), and alanine aminotransferase (ALT) were quantified in terminal plasma samples using the DRI-CHEM NX600V Fujifilm analyzer.

### Statistical analysis

Statistical analysis was conducted using GraphPad (Version 10.0, Prism). Specifically, a one-way ANOVA test (Kruskal-Wallis) was employed for multiple comparisons, and mixed-effect models were utilized to calculate statistical differences for tumor volume and body weight. Kaplan-Meier graphs with log-rank tests were employed to compare differences in survival.

## Results

### Comparative efficacy of ^177^Lu-SN201

**Fig. 1:**
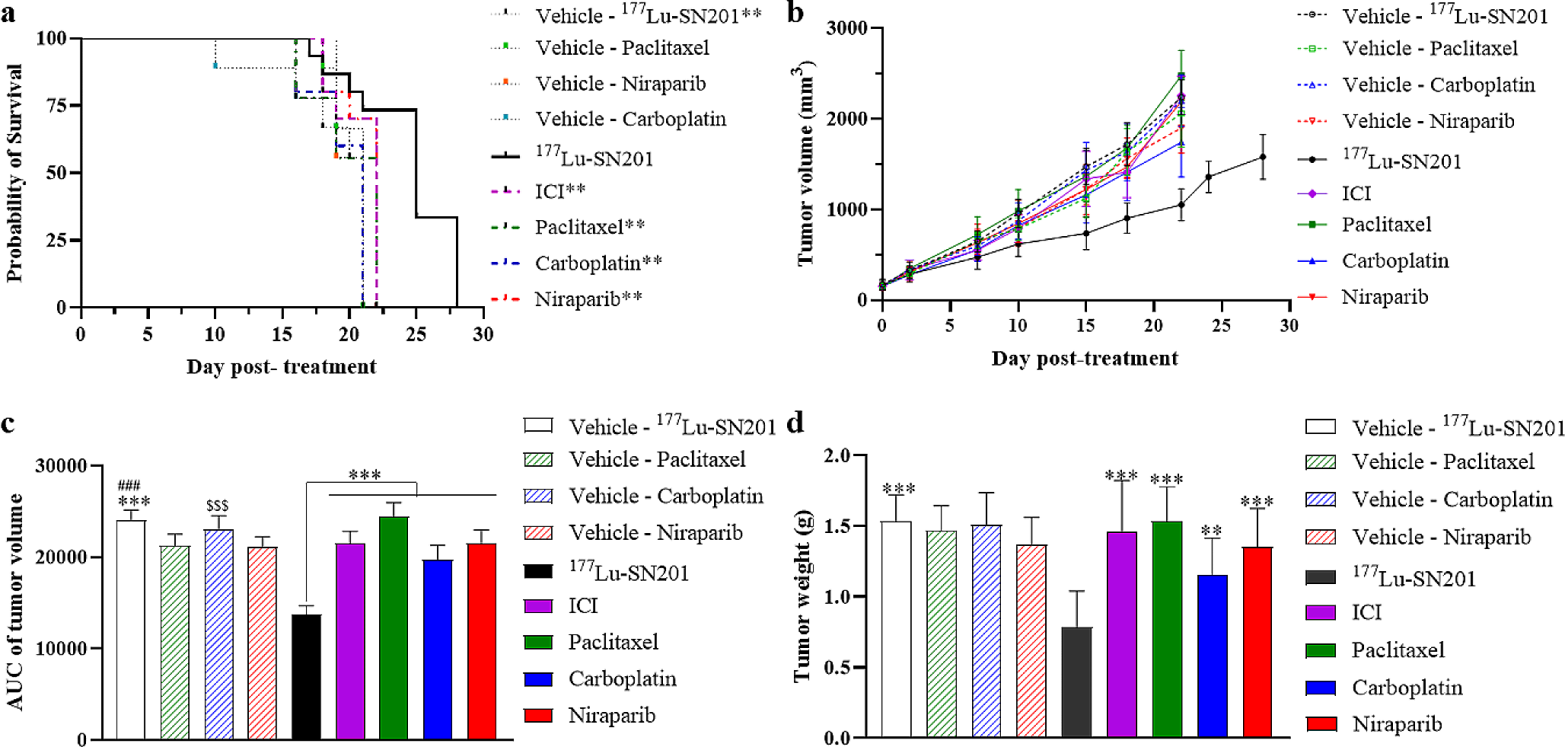
Comparison of therapeutic efficacy among treatments in murine xenograft model. **(a)** Survival curves showing prolonged survival with ^177^Lu-SN201 compared to ICI, paclitaxel, carboplatin, and niraparib. **(b)** Tumor growth inhibition assessed by tumor volume measurements. **(c)** Area under the curve (AUC) of tumor volume up to day 22. **(d)** Tumor weight analysis further confirms the superior efficacy of ^177^Lu-SN201. Asterisks denote statistically significant differences compared to the ^177^Lu-SN201 treatment group (**: *p* < 0.01, ***: *p* < 0.001). # and $ indicate significant differences compared to the ICI and Carboplatin groups, respectively (both ### and $$$: *P* < 0.001). Data are presented as mean ± standard error of the mean (SEM)

In this study, we compared the antitumor efficacy of ^177^Lu-SN201 with other cancer drugs, including ICI, Paclitaxel, Niraparib, and Carboplatin in a 4T1 orthotropic model. Mice were divided into 9 groups, including vehicle controls and treatment groups for each drug. Efficacy was assessed based on survival, tumor weight, and tumor volume. Our results revealed that ^177^Lu-SN201 significantly improved survival compared to the corresponding vehicle (*P* < 0.01) and prolonged survival compared to other drugs (*P* < 0.01 for all comparisons) (Fig. 1a). From visual inspection of the curves, it was observed that ^177^Lu-SN201 reduced tumor volume compared to the vehicles and other drugs (Fig. 1b). To statistically compare tumor volumes, the area under the curve (AUC) up to day 22 was calculated, and statistical significance was determined using One-way ANOVA. ^177^Lu-SN201, ICI, and Carboplatin significantly reduced tumor growth compared to corresponding vehicle treatments (*P* < 0.001) (Fig. 1c). Only ^177^Lu-SN201 significantly reduced tumor weight compared to the corresponding vehicle (*P* < 0.0001) (Fig. 1d). Notably, ^177^Lu-SN201-treated groups exhibited significantly lower tumor volumes and weights, with tumor weight being notably lower in the ^177^Lu-SN201 group compared to other treatment groups (*P* < 0.01 for all comparisons) (Fig. 1c & d).

### Body weight

**Fig. 2:**
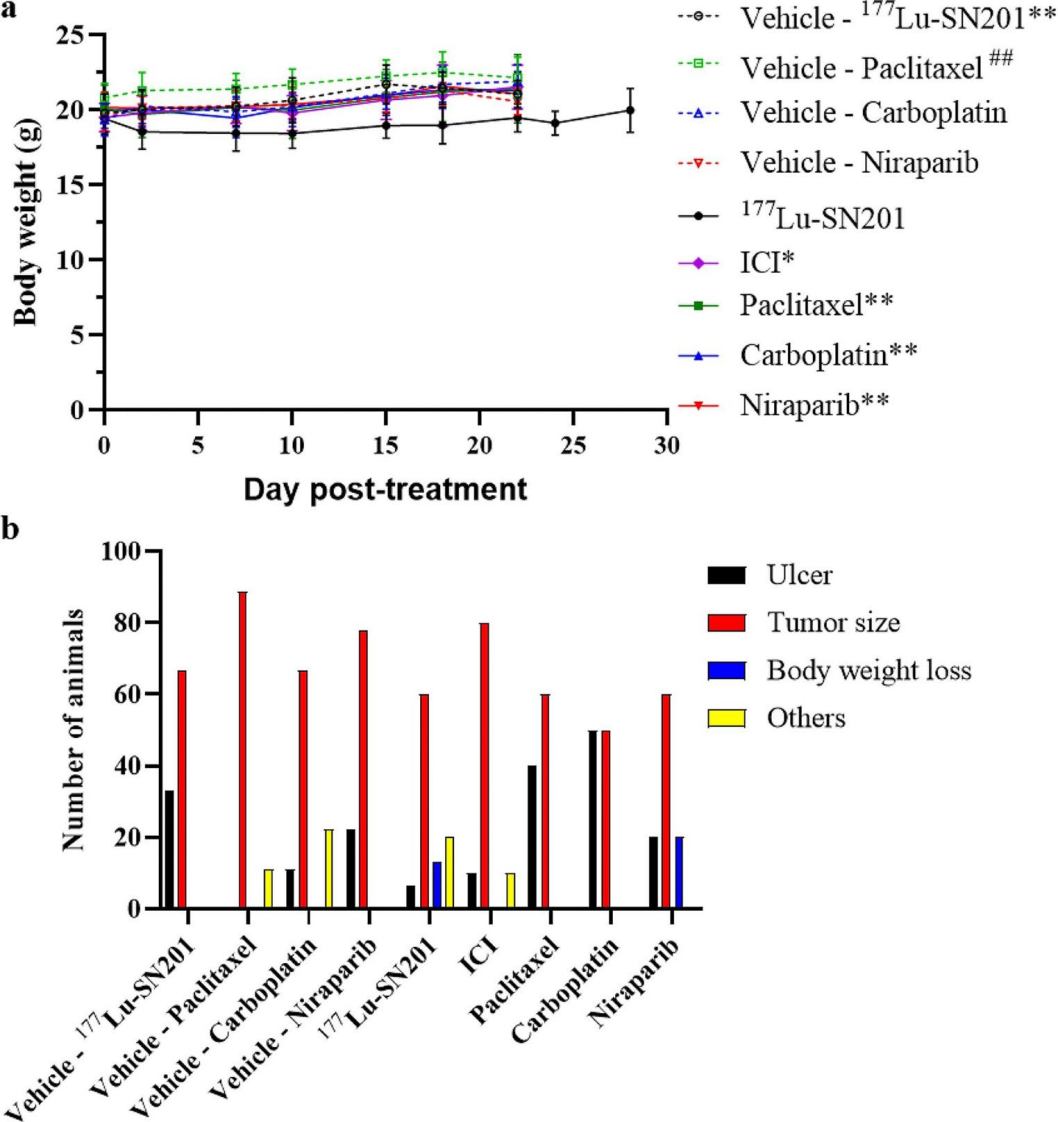
**(a)** Monitoring of mice body weight throughout the observation period. Asterisks denote significant differences between the ^177^Lu-SN201 group and other treatment groups (*: *P* > 0.05, **: *P* < 0.01). The hash sign indicates significant differences compared to the Paclitaxel group (#: *P* < 0.05). **(b)** Percentage distribution of termination causes among treatment groups

Body weight measurements were recorded twice weekly over a period of 28 days. Analysis revealed that mice receiving ^177^Lu-SN201 (*P* < 0.01) and Paclitaxel (*P* < 0.01) exhibited significantly lower body weights compared to their corresponding control groups. Furthermore, mice receiving ^177^Lu-SN201 displayed lower body weights compared to those receiving other treatments (Fig. 2a). Although significant weight lost was observed in mice received ^177^Lu-SN201, the majority of mice were terminated due to tumor reaching humane endpoint (Fig. 2b).

### Toxicity analysis

In light of observed toxicity with ^177^Lu-SN01 treatment at high doses [4], we conducted assessments to monitor kidney and liver function in the treated animals. Liver function was evaluated through measurements of glutamate pyruvate alanine aminotransferase (GPT) and alanine amino transferase (ALP) levels in plasma samples, while kidney function was assessed by testing blood urea nitrogen (BUN) and creatinine levels in plasma samples. The samples were collected at the time of termination. Given carboplatin’s known nephrotoxic effects [18], the carboplatin group was also included in the analysis. Since the ^177^Lu-SN201 vehicle was Ringer’s buffer, which was not expected to adversely affect liver and kidney function, only the carboplatin vehicle (5% DMSO) was assessed. Analysis using the Kruskal-Wallis test revealed no significant differences in serum ALP, GPT, and creatinine levels among the groups. However, serum BUN levels were significantly elevated in the ^177^Lu-SN201-treated group (21.77 U/L) compared to the carboplatin-treated group (16.93 U/L); *P* < 0.05, with no significant difference observed compared to the vehicle. Importantly, serum levels of ALP, GPT, creatinine, and BUN all remained within the reference range [19].

**Fig. 3:**
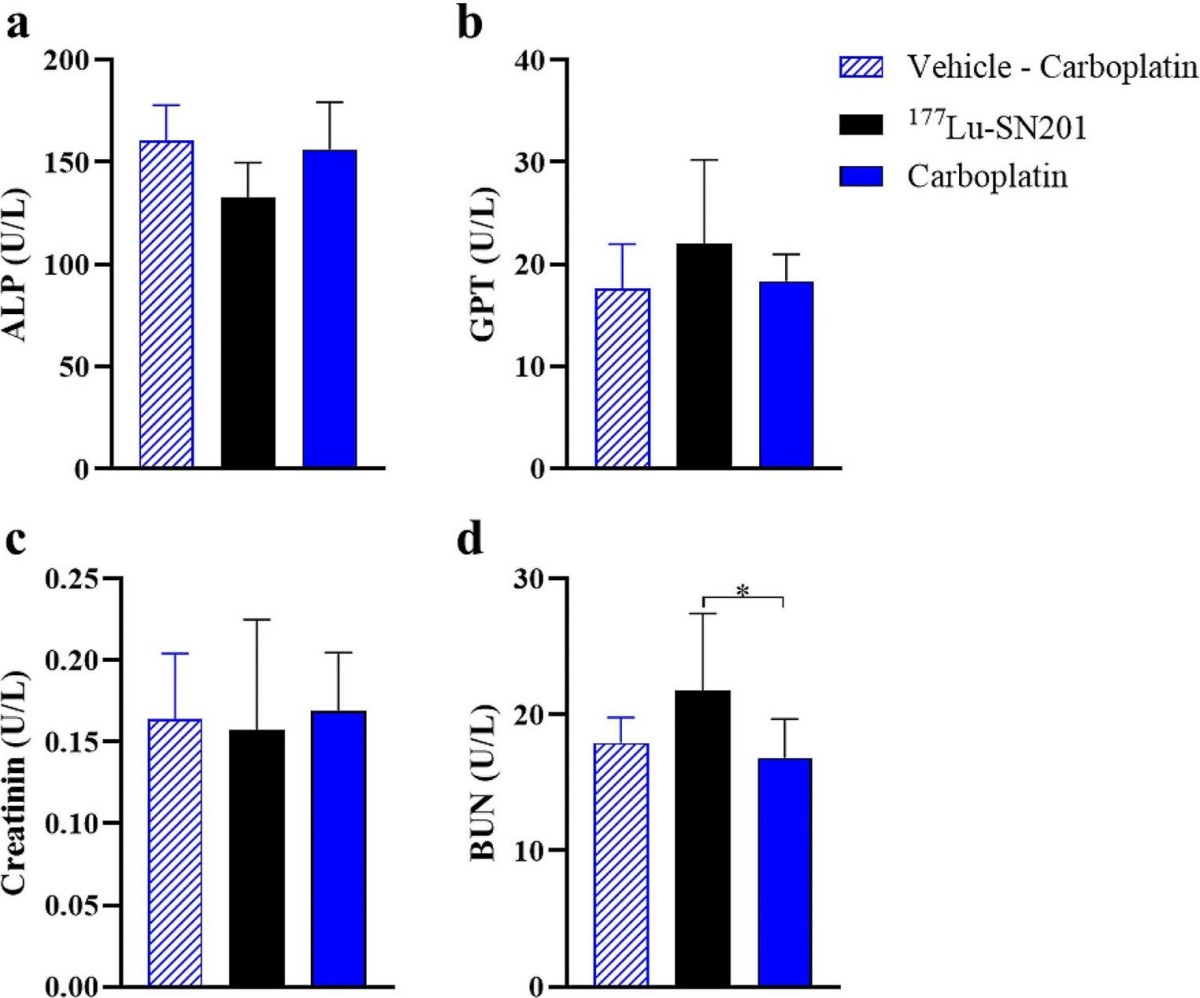
Clinical chemistry analysis depicting the levels of ALP (alkaline phosphatase) **(a)**, GPT (alanine transaminase) **(b)**, creatinine **(c)**, and BUN (blood urea nitrogen) **(d)** in terminal plasma samples

## Discussion

Our study aimed to assess the antitumor efficacy and potential toxicity of ^177^Lu-SN201 compared to other standard cancer drugs in a murine model. Our findings indicate that ^177^Lu-SN201-treated mice exhibited significantly longer survival times and reduced tumor burden compared to both vehicle controls and other treatment groups, suggesting that ^177^Lu-SN201 may exert more effective anticancer activity than some conventional drugs, at least in this very aggressive tumor model. These results are consistent with previous studies demonstrating the promising therapeutic potential of ^177^Lu-SN201 in various cancer types.

A noteworthy observation in our study was the reduction in body weight among mice receiving ^177^Lu-SN201, indicating a potential concern for toxicity. However, clinical chemistry analysis revealed that ^177^Lu-SN201 was still well-tolerated, with liver and kidney function markers remaining within the reference range. It is important to note that while changes in body weight can serve as an indicator of toxicity, they must be interpreted alongside comprehensive toxicity assessments to accurately evaluate the safety profile of a therapeutic agent. In this case we suspect that myelotoxicity will be the dose limiting factor in humans and we appreciate how difficult it is to predict the acceptable clinical dose from mouse data and are awaiting results from the current clinical study.

## Conclusion

In conclusion, our study demonstrates the potential of ^177^Lu-SN201 as an effective anticancer treatment, evidenced by significantly prolonged survival and reduced tumor burden in the aggressive 4T1 murine breast cancer model. It is considered a model of triple negative breast cancer, which is very difficult to treat with the drugs currently available [20]. In particular, we show with high statistical significance that ^177^Lu-SN201 is superior at slowing the tumor growth and prolonging the life time compared to the commonly used cancer drugs paclitaxel, niraparib, carboplatin, and the combination of the immune check-point inhibitors anti PD-1 and anti-CTLA-4.

The treatment is reasonably well-tolerated, as indicated by clinical chemistry analysis, despite some weight loss. Overall, ^177^Lu-SN201 presents as a promising therapeutic candidate for cancer treatment, offering hope for improved outcomes in patients, pending continued investigation and clinical development.

## Data Availability

No datasets were generated or analysed during the current study.
